# Supplementary material for: Integrated Interactomics Reveals Novel Protein Associations: The FOXA1-PBX1 Complex as a Case Study
Source: bioRxiv. 2026 Jul 17:2026.07.16.738938. Preprint. [Version 1] doi: 10.64898/2026.07.16.738938 (PMC13404648; doi:10.64898/2026.07.16.738938)
Supplement: Supplement 1 [file NIHPP2026.07.16.738938v1-supplement-1.pdf]

Supplementary Materials

Integrated Interactomics Reveals Novel Protein Associations: The FOXA1-PBX1 Complex as a Case Study

Zhi Chen, Istvan Szepesi-Nagy, Qingyue Zhang, Srushti Kittane, Yangzhenyu Gao, Juliana Ortiz-Pacheco, Ethan Lane, Justin Hatcher, Jeffrey Estrada, Manor Askenazi, Lan Huang, Beatrix Ueberheide, Ning Zheng, Eneda Toska, Gergely Rona, Michele Pagano

Outline

Supplementary Figures:

Figure S1 corresponds to Figure 1
Figure S2 corresponds to Figures 1 and 2
Figure S3 corresponds to Figure 2
Figure S4 corresponds to Figures 1 and 2
Figure S5 corresponds to Figures 1 and 2
Figure S6 corresponds to Figure 2
Figure S7 corresponds to Figure 3
Figure S8 corresponds to Figure 4

Supplementary Methods

SUPPLEMENTARY FIGURES

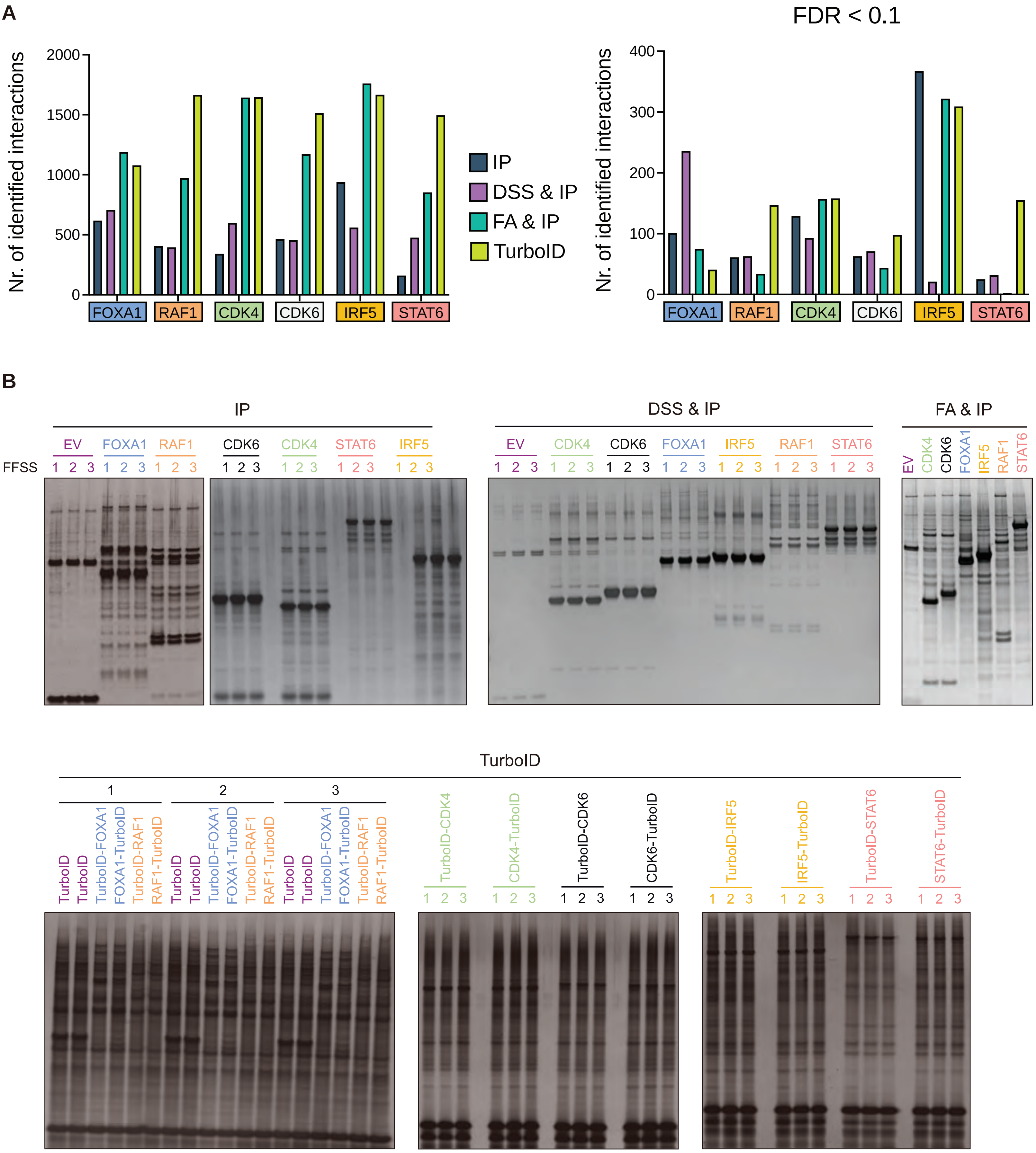
A
FDR < 0.1
Nr. of identified interactions
IP
DSS & IP
FA & IP
TurboID
FOXA1
RAF1
CDK4
CDK6
IRF5
STAT6
B
IP
DSS & IP
FA & IP
FFSS
EV
TurboID
TurboID-FOXA1
FOXA1-TurboID
TurboID-RAF1
RAF1-TurboID
TurboID-CDK4
CDK4-TurboID
TurboID-CDK6
CDK6-TurboID
TurboID-IRF5
IRF5-TurboID
TurboID-STAT6
STAT6-TurboID

Figure S1

Figure S1. Global overview of identified, known and recovered interactions and technical validation of affinity purification approaches.

(A) Bar plots summarize, for each bait protein and affinity purification technique, the total number of detected interaction partners (left) and the subset of significantly enriched interactions (right), defined by a false discovery rate (FDR) < 0.1.

(B) Representative silver staining analyses demonstrating the technical performance and reproducibility of each affinity purification strategy.

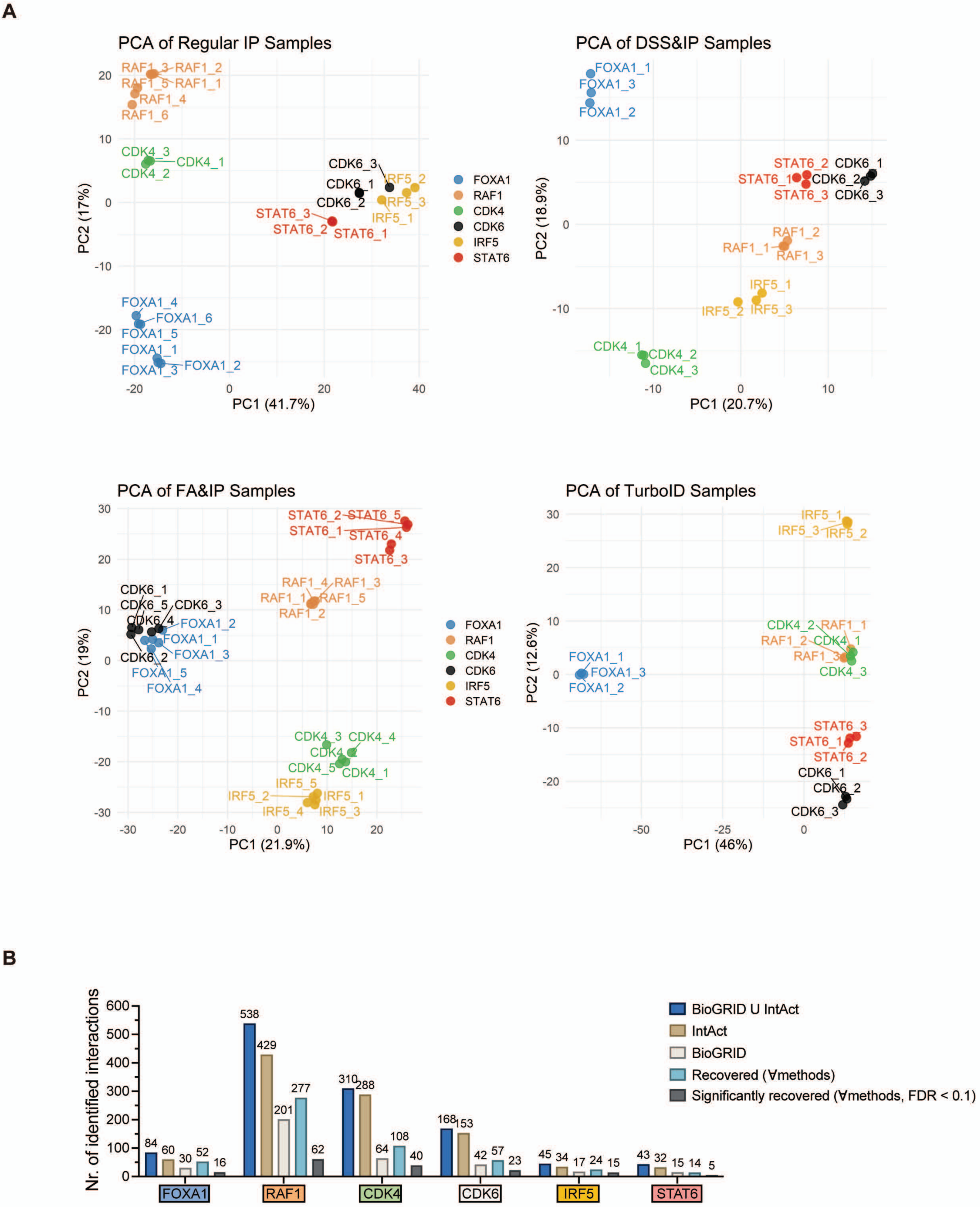
A
PCA of Regular IP Samples
PC1 (41.7%)
PC2 (17%)
PCA of DSS&IP Samples
PC1 (20.7%)
PC2 (18.9%)
PCA of FA&IP Samples
PC1 (21.9%)
PC2 (19%)
PCA of TurboID Samples
PC1 (46%)
PC2 (12.6%)
FOXA1
RAF1
CDK4
CDK6
IRF5
STAT6
B
Nr. of identified interactions
BioGRID U IntAct
IntAct
BioGRID
Recovered (∀methods)
Significantly recovered (∀methods, FDR < 0.1)
FOXA1: 84, 60, 30, 52, 16
RAF1: 538, 429, 201, 277, 62
CDK4: 310, 288, 64, 108, 40
CDK6: 168, 153, 42, 57, 23
IRF5: 45, 34, 17, 24, 15
STAT6: 43, 32, 15, 14, 5

Figure S2

Figure S2. Technical replicates represent high methodological validity of each mass spectrometry run for all baits per technique.

(A) Principal component analysis (PCA) plots illustrating the clustering of technical replicates from mass spectrometry analyses for all bait proteins across each affinity purification method: conventional immunoprecipitation (IP); DSS crosslinking followed by IP (DSS & IP); formaldehyde crosslinking followed by IP (FA & IP); and TurboID-based proximity labeling. Tight clustering of technical replicates demonstrates high methodological reproducibility for each technique.

(B) Bar plots summarize the number of known interactors and recovered proteins for each bait. Bar heights depict the number of interactors in five categories: (1) the union of BioGRID and IntAct, (2) IntAct alone, (3) BioGRID alone, (4) the total number of interactors recovered across all four AP-MS experiments, and (5) the subset of these recovered interactors that are significant hits.

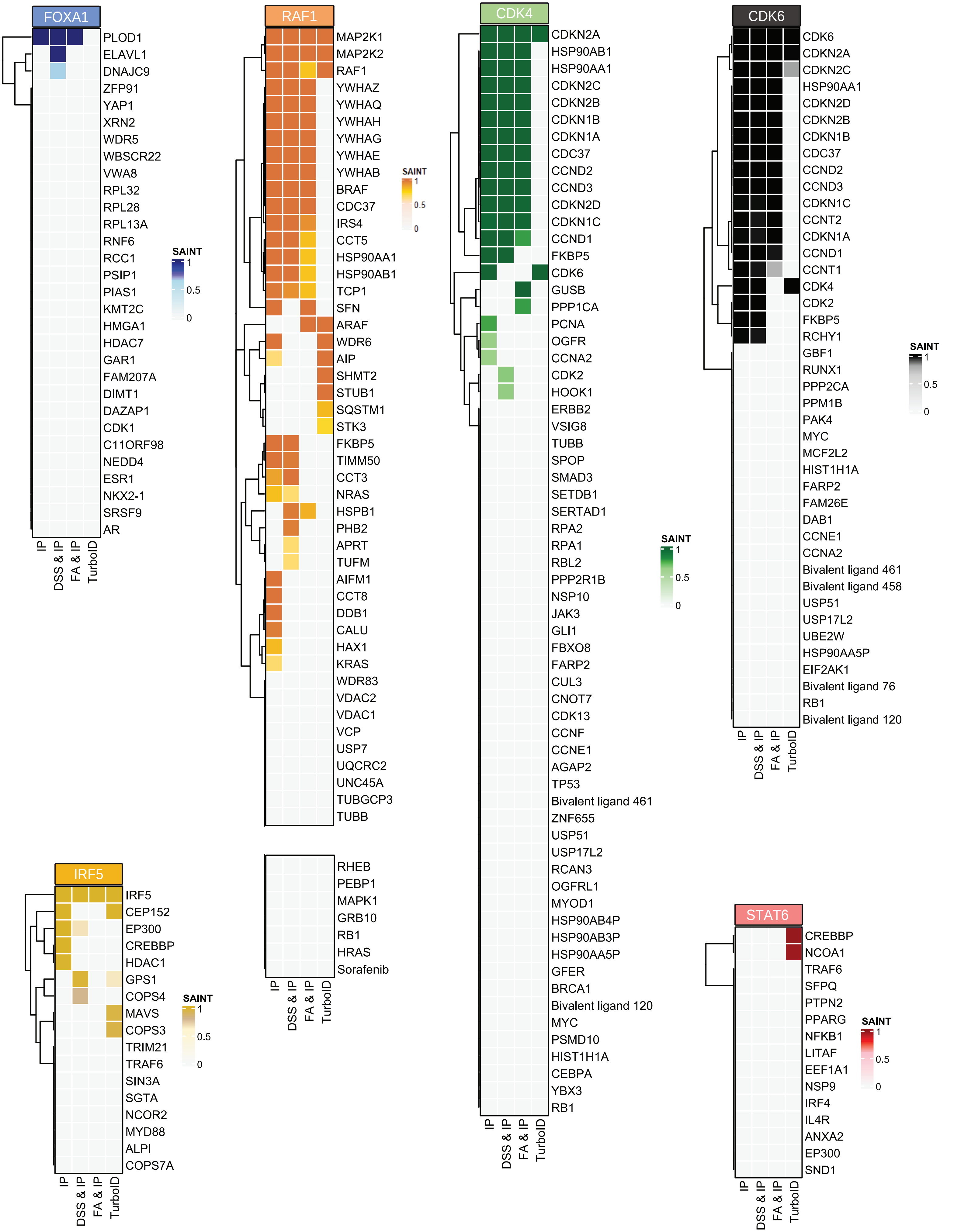
FOXA1
PLOD1
ELAVL1
DNAJC9
ZFP91
YAP1
XRN2
WDR5
WBSCR22
VWA8
RPL32
RPL28
RPL13A
RNF6
RCC1
PSIP1
PIAS1
KMT2C
HMGA1
HDAC7
GAR1
FAM207A
DIMT1
DAZAP1
CDK1
C11ORF98
NEDD4
ESR1
NKX2-1
SRSF9
AR
SAINT
1
0.5
0
IP
DSS & IP
FA & IP
TurboID
RAF1
MAP2K1
MAP2K2
RAF1
YWHAZ
YWHAQ
YWHAH
YWHAG
YWHAE
YWHAB
BRAF
CDC37
IRS4
CCT5
HSP90AA1
HSP90AB1
TCP1
SFN
ARAF
WDR6
AIP
SHMT2
STUB1
SQSTM1
STK3
FKBP5
TIMM50
CCT3
NRAS
HSPB1
PHB2
APRT
TUFM
AIFM1
CCT8
DDB1
CALU
HAX1
KRAS
WDR83
VDAC2
VDAC1
VCP
USP7
UQCRC2
UNC45A
TUBGCP3
TUBB
RHEB
PEBP1
MAPK1
GRB10
RB1
HRAS
Sorafenib
SAINT
1
0.5
0
IP
DSS & IP
FA & IP
TurboID
CDK4
CDKN2A
HSP90AB1
HSP90AA1
CDKN2C
CDKN2B
CDKN1B
CDKN1A
CDC37
CCND2
CCND3
CDKN2D
CDKN1C
CCND1
FKBP5
CDK6
GUSB
PPP1CA
PCNA
OGFR
CCNA2
CDK2
HOOK1
ERBB2
VSIG8
TUBB
SPOP
SMAD3
SETDB1
SERTAD1
RPA2
RPA1
RBL2
PPP2R1B
NSP10
JAK3
GLI1
FBXO8
FARP2
CUL3
CNOT7
CDK13
CCNF
CCNE1
AGAP2
TP53
Bivalent ligand 461
ZNF655
USP51
USP17L2
RCAN3
OGFRL1
MYOD1
HSP90AB4P
HSP90AB3P
HSP90AA5P
GFER
BRCA1
Bivalent ligand 120
MYC
PSMD10
HIST1H1A
CEBPA
YBX3
RB1
SAINT
1
0.5
0
IP
DSS & IP
FA & IP
TurboID
CDK6
CDK6
CDKN2A
CDKN2C
HSP90AA1
CDKN2D
CDKN2B
CDKN1B
CDC37
CCND2
CCND3
CDKN1C
CCNT2
CDKN1A
CCND1
CCNT1
CDK4
CDK2
FKBP5
RCHY1
GBF1
RUNX1
PPP2CA
PPM1B
PAK4
MYC
MCF2L2
HIST1H1A
FARP2
FAM26E
DAB1
CCNE1
CCNA2
Bivalent ligand 461
Bivalent ligand 458
USP51
USP17L2
UBE2W
HSP90AA5P
EIF2AK1
Bivalent ligand 76
RB1
Bivalent ligand 120
SAINT
1
0.5
0
IP
DSS & IP
FA & IP
TurboID
IRF5
IRF5
CEP152
EP300
CREBBP
HDAC1
GPS1
COPS4
MAVS
COPS3
TRIM21
TRAF6
SIN3A
SGTA
NCOR2
MYD88
ALPI
COPS7A
SAINT
1
0.5
0
IP
DSS & IP
FA & IP
TurboID
STAT6
CREBBP
NCOA1
TRAF6
SFPQ
PTPN2
PPARG
NFKB1
LITAF
EEF1A1
NSP9
IRF4
IL4R
ANXA2
EP300
SND1
SAINT
1
0.5
0
IP
DSS & IP
FA & IP
TurboID

Figure S3

Figure S3. Recovery of known BioGRID interactions across affinity purification techniques. Heatmaps displaying BioGRID-curated interaction partners identified for each bait protein across all affinity purification methods. Only proteins detected by mass spectrometry in the corresponding experiments are shown, enabling direct comparison of each technique's ability to recapitulate previously reported interactions. Color intensity represents the corresponding SAINT score for each interaction.

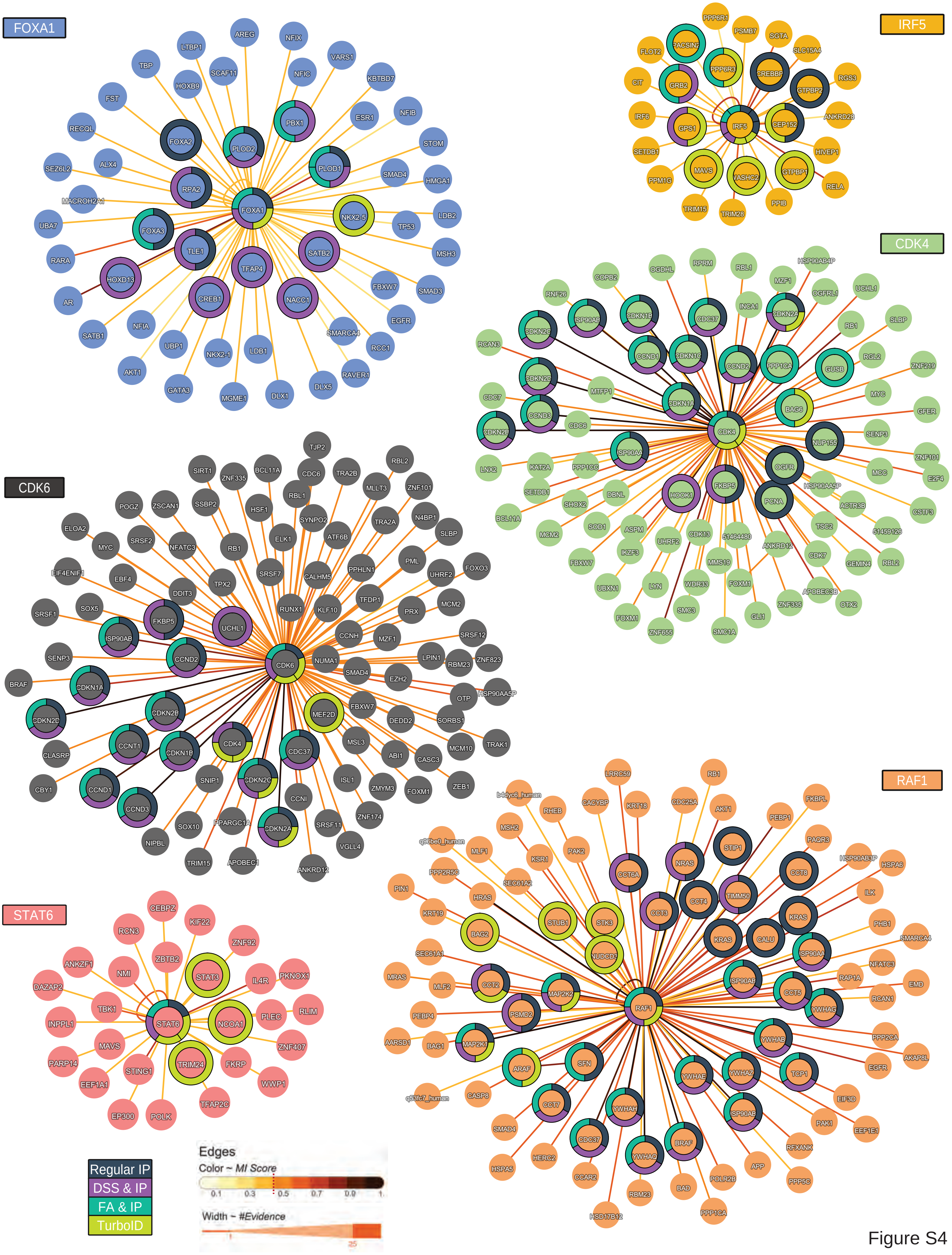
FOXA1
AREG
NFIX
LTBP1
VARS1
TBP
SCAF11
NFIC
KBTBD7
HOXB9
FST
PBX1
NFIB
ESR1
RECQL
FOXA2
PLOD2
STOM
ALX4
SEZ6L2
PLOD1
SMAD4
HMGA1
RPA2
MACROH2A1
FOXA1
NKX2-5
LDB2
UBA7
FOXA3
TP53
TLE1
SATB2
MSH3
RARA
TFAP4
HOXD13
FBXW7
SMAD3
AR
CREB1
NACC1
EGFR
SATB1
NFIA
SMARCA4
UBP1
NKX2-1
LDB1
RCC1
AKT1
RAVER1
DLX5
GATA3
MGME1
DLX1
IRF5
PPP6R1
PACSIN2
PSMB7
SGTA
FLOT2
SLC15A4
PPP6R3
CREBBP
RGS3
CIT
GRB2
GTPBP2
IRF6
ANKRD28
GPS1
IRF5
CEP152
SETDB1
HIVEP1
MAVS
GTPBP1
PPM1G
VASHC2
RELA
PPIB
TRIM15
TRIM28
CDK4
RPRM
HSP90AB4P
OGDHL
RBL1
COPB2
MZF1
UCHL1
RNF26
OGFRL1
CDKN1B
INCA1
HSP90AB
CDKN2A
CDC37
RB1
SLBP
CDKN2C
RCAN3
CCND1
CDKN1C
CCND2
PPP1CA
GUSB
RGL2
ZNF219
CDKN2D
MTFP1
MYC
CDC7
CDKN1A
BAG6
GFER
CCND3
CDC6
CDKN2B
CDK4
SENP3
NUP155
HSP90AA
ZNF101
LNX2
KAT2A
PPP1CC
OGFR
MCC
E2F4
HSP90AA5P
SETDB1
DBNL
FKBP5
HOOK1
ACTR3B
PCNA
CSTF3
SHOX2
BCL11A
TSC2
SOD1
ASPM
51459126
CDK13
51464480
MCM2
UHRF2
ANKRD12
IKZF3
MMS19
CDK7
GEMIN4
RBL2
FBXW7
UBXN1
LYN
WDR33
FOXM1
APOBEC3B
OTX2
SMC3
ZNF335
GLI1
FOXM1
ZNF655
SMC1A
CDK6
TJP2
SIRT1
BCL11A
RBL2
ZNF335
CDC6
TRA2B
MLLT3
ZNF101
RBL1
POGZ
ZSCAN1
SSBP2
HSF1
ELOA2
SYNPO2
N4BP1
TRA2A
SLBP
MYC
SRSF2
NFATC3
RB1
ELK1
ATF6B
PML
EIF4ENIF1
EBF4
UHRF2
FOXO3
SRSF7
CALHM5
PPHLN1
TPX2
DDIT3
TFDP1
MCM2
SOX5
SRSF1
RUNX1
KLF10
PRX
FKBP5
UCHL1
HSP90AB
CCNH
MZF1
SRSF12
LPIN1
SENP3
CCND2
NUMA1
RBM23
ZNF823
CDK6
SMAD4
BRAF
EZH2
CDKN1A
HSP90AA5P
OTP
FBXW7
MEF2D
CDKN2D
CDKN2B
DEDD2
SORBS1
CDK4
TRAK1
CCNT1
MSL3
CLASRP
CDC37
MCM10
CDKN1B
ABI1
CASC3
SNIP1
CDKN2C
ISL1
CBY1
CCND1
ZMYM3
FOXM1
ZEB1
CCNI
CCND3
ZNF174
PPARGC1A
SOX10
SRSF11
CDKN2A
NIPBL
VGLL4
TRIM15
APOBEC1
ANKRD12
RAF1
LRRC59
RB1
b4dyc6_human
CACYBP
KRT18
CDC25A
AKT1
FKBPL
RHEB
PEBP1
MSH2
PAQR3
q96be0_human
PAK2
MLF1
KSR1
STIP1
HSP90AB3P
CCT6A
NRAS
HSPA6
PPP2R5C
CCT8
SEC61A2
PIN1
CCT4
TIMM50
HRAS
ILK
CCT3
KRT19
STUB1
STK3
KRAS
PHB1
BAG2
KRAS
CALU
SMARCA4
SEC61A1
NUDCD3
HSP90AA
NFATC3
MRAS
RAP1A
EMD
HSP90AB
MLF2
CCT2
MAP2K2
CCT5
RCAN1
PSMD2
RAF1
YWHAG
PEBP4
PPP2CA
YWHAB
AARSD1
BAG1
MAP2K1
AKAP8L
SFN
EGFR
ARAF
YWHAE
YWHAZ
TCP1
q53fc7_human
CASP8
EIF3D
YWHAH
CCT7
HSP90AB
PAK1
EEF1E1
SMAD4
BRAF
RFXANK
CDC37
YWHAQ
HERC2
HSPA5
APP
PPP5C
CCAR2
POLR2B
RBM23
BAD
PPP1CA
HSD17B12
STAT6
CEBPZ
RCN3
KIF22
ZNF92
ANKZF1
ZBTB2
NMI
STAT3
DAZAP2
IL4R
PKNOX1
TBK1
PLEC
RLIM
STAT6
NCOA1
INPPL1
MAVS
ZNF407
PARP14
TRIM24
FKRP
STING1
EEF1A1
WWP1
TFAP2C
EP300
POLK
Regular IP
DSS & IP
FA & IP
TurboID
Edges
Color ~ MI Score
0.1
0.3
0.5
0.7
0.9
1
Width ~ #Evidence
1
25

Figure S4

Figure S4. Benchmarking bait protein interactors using IntAct network analysis.
Graphs show network representation of IntAct database interactors for each bait. First-neighbor interactions are visualized using IntAct's *MI-score* and number of supporting evidence (*#Evidence*). For CDK4 and CDK6, which have large interaction networks, an MI-score cutoff ≥ 0.4 was applied for clarity. Donut coloring highlights which techniques in our study successfully captured each interactor, providing a benchmark of experimental performance.

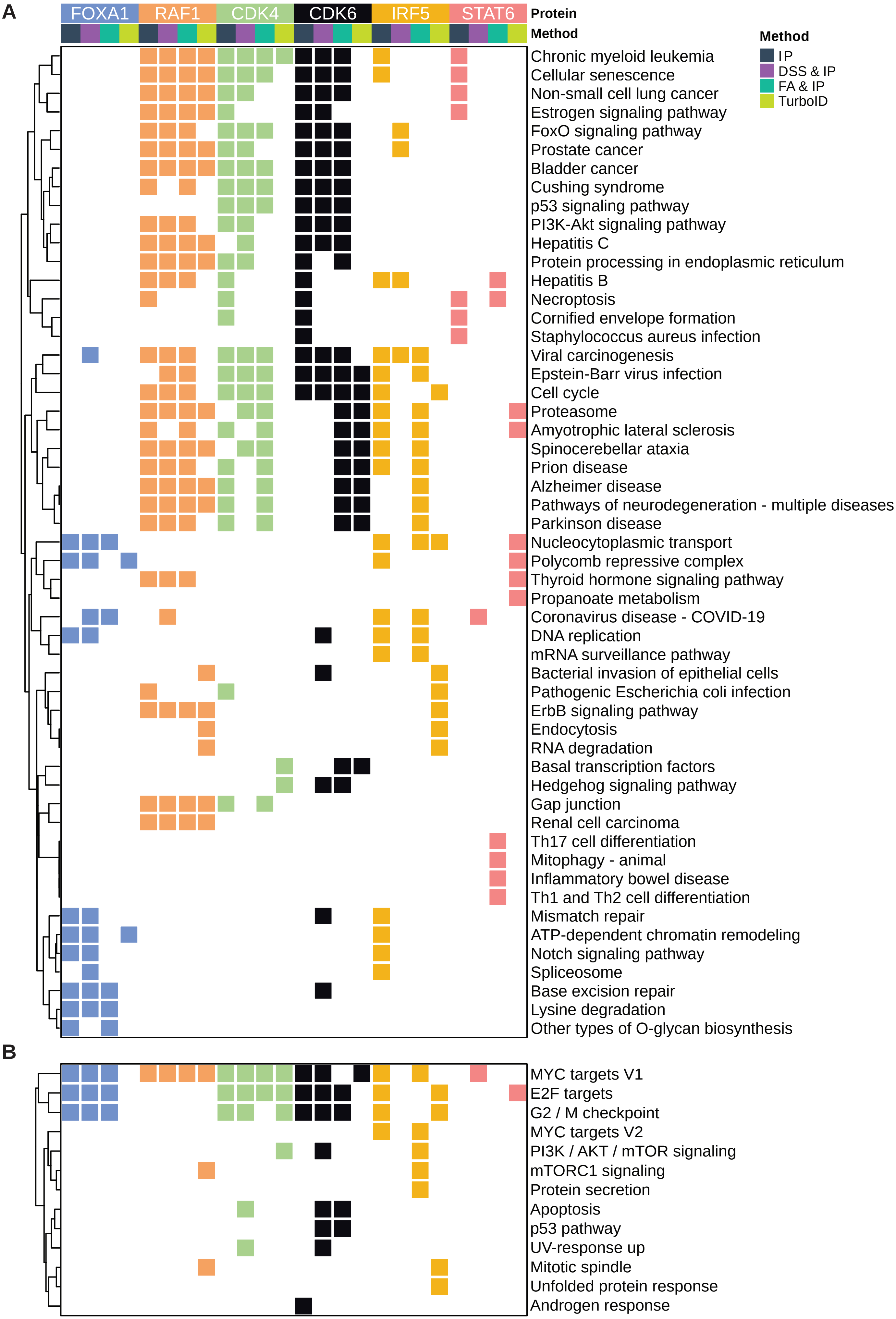
A
FOXA1
RAF1
CDK4
CDK6
IRF5
STAT6
Protein
Method
Method
IP
DSS & IP
FA & IP
TurboID
Chronic myeloid leukemia
Cellular senescence
Non-small cell lung cancer
Estrogen signaling pathway
FoxO signaling pathway
Prostate cancer
Bladder cancer
Cushing syndrome
p53 signaling pathway
PI3K-Akt signaling pathway
Hepatitis C
Protein processing in endoplasmic reticulum
Hepatitis B
Necroptosis
Cornified envelope formation
Staphylococcus aureus infection
Viral carcinogenesis
Epstein-Barr virus infection
Cell cycle
Proteasome
Amyotrophic lateral sclerosis
Spinocerebellar ataxia
Prion disease
Alzheimer disease
Pathways of neurodegeneration - multiple diseases
Parkinson disease
Nucleocytoplasmic transport
Polycomb repressive complex
Thyroid hormone signaling pathway
Propanoate metabolism
Coronavirus disease - COVID-19
DNA replication
mRNA surveillance pathway
Bacterial invasion of epithelial cells
Pathogenic Escherichia coli infection
ErbB signaling pathway
Endocytosis
RNA degradation
Basal transcription factors
Hedgehog signaling pathway
Gap junction
Renal cell carcinoma
Th17 cell differentiation
Mitophagy - animal
Inflammatory bowel disease
Th1 and Th2 cell differentiation
Mismatch repair
ATP-dependent chromatin remodeling
Notch signaling pathway
Spliceosome
Base excision repair
Lysine degradation
Other types of O-glycan biosynthesis
B
MYC targets V1
E2F targets
G2 / M checkpoint
MYC targets V2
PI3K / AKT / mTOR signaling
mTORC1 signaling
Protein secretion
Apoptosis
p53 pathway
UV-response up
Mitotic spindle
Unfolded protein response
Androgen response

Figure S5

Figure S5. Comparative functional enrichment mapping across affinity purification techniques.

(A) Heatmap showing the union of the top five enriched KEGG pathways for each bait protein identified by each affinity purification technique. Pathways ranking within the top five in any condition were included.

(B) Heatmap showing the union of all significantly enriched (*adj.p* < 0.05) MSigDB Hallmark pathways for each bait protein identified by each affinity purification technique.

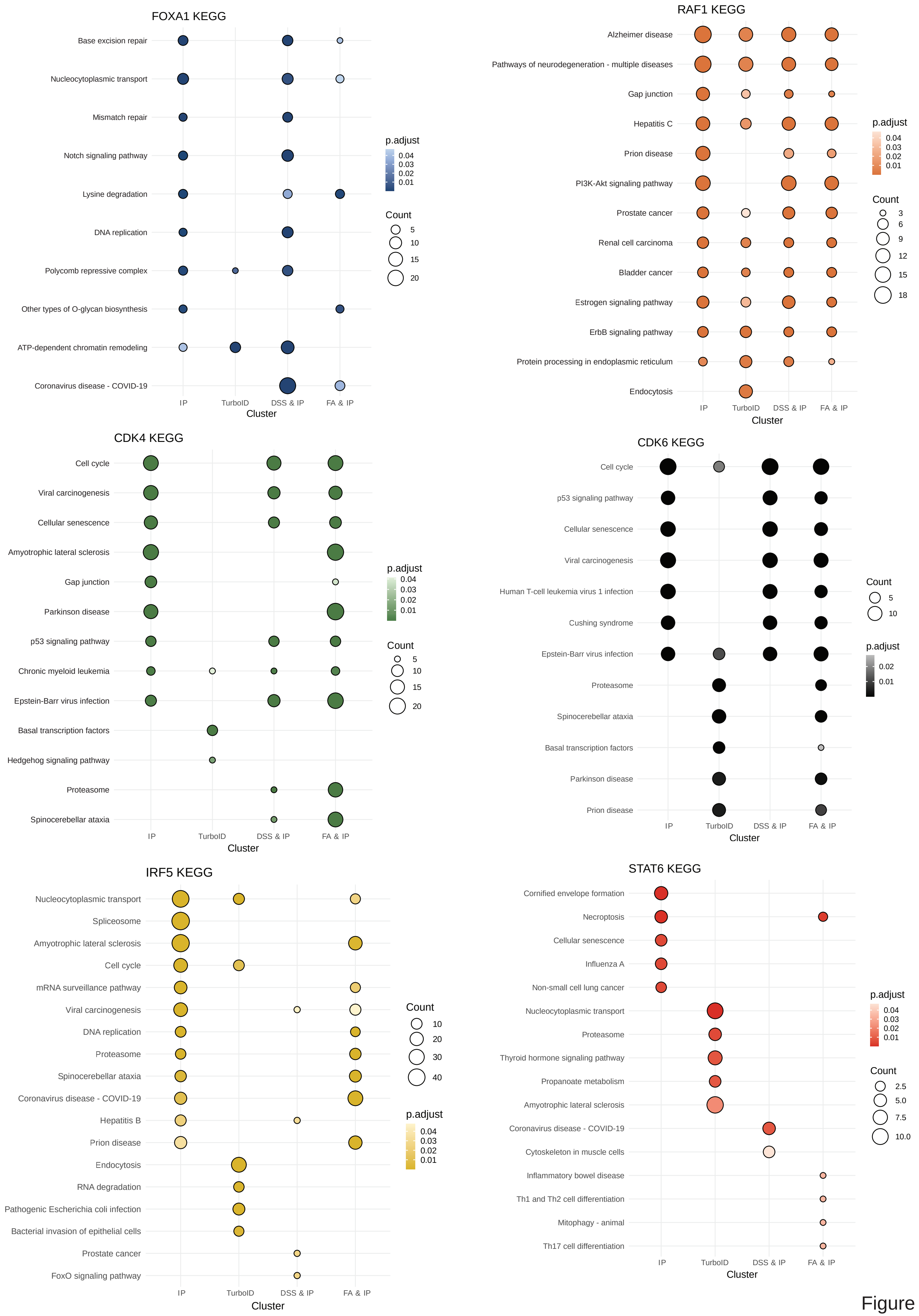
FOXA1 KEGG
Base excision repair
Nucleocytoplasmic transport
Mismatch repair
Notch signaling pathway
Lysine degradation
DNA replication
Polycomb repressive complex
Other types of O-glycan biosynthesis
ATP-dependent chromatin remodeling
Coronavirus disease - COVID-19
IP
TurboID
DSS & IP
FA & IP
Cluster
p.adjust
0.04
0.03
0.02
0.01
Count
5
10
15
20
RAF1 KEGG
Alzheimer disease
Pathways of neurodegeneration - multiple diseases
Gap junction
Hepatitis C
Prion disease
PI3K-Akt signaling pathway
Prostate cancer
Renal cell carcinoma
Bladder cancer
Estrogen signaling pathway
ErbB signaling pathway
Protein processing in endoplasmic reticulum
Endocytosis
IP
TurboID
DSS & IP
FA & IP
Cluster
p.adjust
0.04
0.03
0.02
0.01
Count
3
6
9
12
15
18
CDK4 KEGG
Cell cycle
Viral carcinogenesis
Cellular senescence
Amyotrophic lateral sclerosis
Gap junction
Parkinson disease
p53 signaling pathway
Chronic myeloid leukemia
Epstein-Barr virus infection
Basal transcription factors
Hedgehog signaling pathway
Proteasome
Spinocerebellar ataxia
IP
TurboID
DSS & IP
FA & IP
Cluster
p.adjust
0.04
0.03
0.02
0.01
Count
5
10
15
20
CDK6 KEGG
Cell cycle
p53 signaling pathway
Cellular senescence
Viral carcinogenesis
Human T-cell leukemia virus 1 infection
Cushing syndrome
Epstein-Barr virus infection
Proteasome
Spinocerebellar ataxia
Basal transcription factors
Parkinson disease
Prion disease
IP
TurboID
DSS & IP
FA & IP
Cluster
Count
5
10
p.adjust
0.02
0.01
IRF5 KEGG
Nucleocytoplasmic transport
Spliceosome
Amyotrophic lateral sclerosis
Cell cycle
mRNA surveillance pathway
Viral carcinogenesis
DNA replication
Proteasome
Spinocerebellar ataxia
Coronavirus disease - COVID-19
Hepatitis B
Prion disease
Endocytosis
RNA degradation
Pathogenic Escherichia coli infection
Bacterial invasion of epithelial cells
Prostate cancer
FoxO signaling pathway
IP
TurboID
DSS & IP
FA & IP
Cluster
Count
10
20
30
40
p.adjust
0.04
0.03
0.02
0.01
STAT6 KEGG
Cornified envelope formation
Necroptosis
Cellular senescence
Influenza A
Non-small cell lung cancer
Nucleocytoplasmic transport
Proteasome
Thyroid hormone signaling pathway
Propanoate metabolism
Amyotrophic lateral sclerosis
Coronavirus disease - COVID-19
Cytoskeleton in muscle cells
Inflammatory bowel disease
Th1 and Th2 cell differentiation
Mitophagy - animal
Th17 cell differentiation
IP
TurboID
DSS & IP
FA & IP
Cluster
p.adjust
0.04
0.03
0.02
0.01
Count
2.5
5.0
7.5
10.0

Figure S6

Figure S6. Integrated KEGG pathway enrichment across affinity purification techniques. Dot plot summarizing KEGG pathway enrichment results merged by bait protein across all affinity purification methods. For each purification technique, the top five enriched KEGG pathways were identified, and the combined non-redundant set of pathways was used for visualization. Dot color represents the adjusted p value (p.adjust), while dot size corresponds to the number of genes contributing to each enriched pathway.

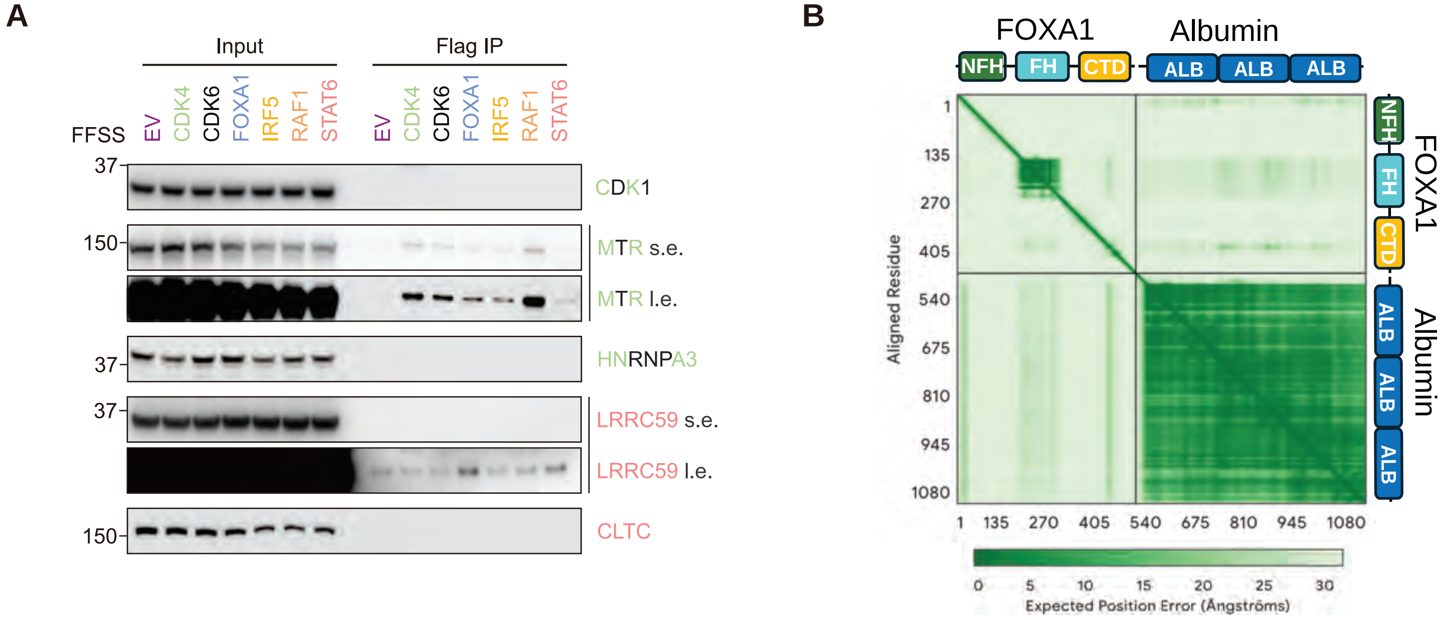

A
Input
Flag IP
FFSS
EV
CDK4
CDK6
FOXA1
IRF5
RAF1
STAT6
37
150
37
37
150
CDK1
MTR s.e.
MTR l.e.
HNRNPA3
LRRC59 s.e.
LRRC59 l.e.
CLTC
B
FOXA1
Albumin
NFH
FH
CTD
ALB
Aligned Residue
1
135
270
405
540
675
810
945
1080
0
5
10
15
20
25
30
Expected Position Error (Ångströms)

Figure S7

Figure S7. AlphaFold3 predicts FOXA1-PBX1a direct interaction: The expected position error, measured in Ångströms, depicts the confidence of an interaction. Regions of high confidence reflect interactions between structured domains.

(A) HEK293T cells were transfected with either an empty vector (EV) or the indicated twin-FLAG- and twin-Strep-tagged proteins. Twenty-four hours after transfection, cells were harvested for immunoprecipitation (IP) and immunoblotting. Colors denote the corresponding bait proteins and their associated interaction hits. Alternating green and black labels indicate proteins identified as shared candidate interactors of both CDK4 and CDK6. s.e., short exposure; l.e., long exposure.

(B) Depicts a nonspecific, low confidence interaction between FOXA1, a cytosolic and nuclear protein, with Albumin, a secreted protein that will not interact with FOXA1. The PAE of the FOXA1-Albumin interaction is relatively high in the forkhead and albumin domains indicating low positional confidence of the domain interfaces. The low positional confidence indicates there is no direct binding between FOXA1 and Albumin.

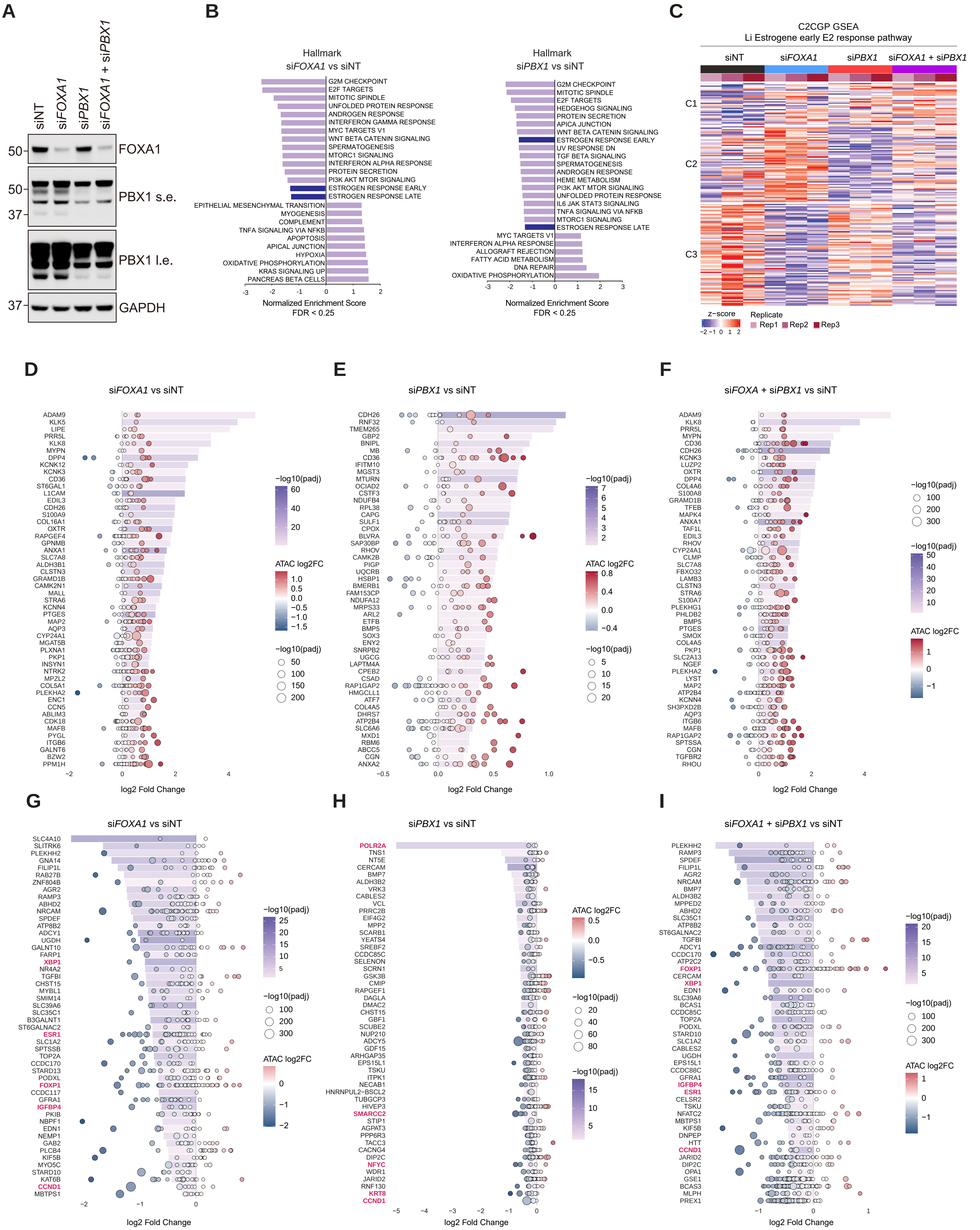
A
siNT
siFOXA1
siPBX1
siFOXA1 + siPBX1
50
FOXA1
50
37
PBX1 s.e.
PBX1 l.e.
37
GAPDH
B
Hallmark
siFOXA1 vs siNT
G2M CHECKPOINT
E2F TARGETS
MITOTIC SPINDLE
UNFOLDED PROTEIN RESPONSE
ANDROGEN RESPONSE
INTERFERON GAMMA RESPONSE
MYC TARGETS V1
WNT BETA CATENIN SIGNALING
SPERMATOGENESIS
MTORC1 SIGNALING
INTERFERON ALPHA RESPONSE
PROTEIN SECRETION
PI3K AKT MTOR SIGNALING
ESTROGEN RESPONSE EARLY
ESTROGEN RESPONSE LATE
EPITHELIAL MESENCHYMAL TRANSITION
MYOGENESIS
COMPLEMENT
TNFA SIGNALING VIA NFKB
APOPTOSIS
APICAL JUNCTION
HYPOXIA
OXIDATIVE PHOSPHORYLATION
KRAS SIGNALING UP
PANCREAS BETA CELLS
Normalized Enrichment Score
FDR < 0.25
Hallmark
siPBX1 vs siNT
G2M CHECKPOINT
MITOTIC SPINDLE
E2F TARGETS
HEDGEHOG SIGNALING
PROTEIN SECRETION
APICA JUNCTION
WNT BETA CATENIN SIGNALING
ESTROGEN RESPONSE EARLY
UV RESPONSE DN
TGF BETA SIGNALING
SPERMATOGENESIS
ANDROGEN RESPONSE
HEME METABOLISM
PI3K AKT MTOR SIGNALING
UNFOLDED PROTEIN RESPONSE
IL6 JAK STAT3 SIGNALING
TNFA SIGNALING VIA NFKB
MTORC1 SIGNALING
ESTROGEN RESPONSE LATE
MYC TARGETS V1
INTERFERON ALPHA RESPONSE
ALLOGRAFT REJECTION
FATTY ACID METABOLISM
DNA REPAIR
OXIDATIVE PHOSPHORYLATION
Normalized Enrichment Score
FDR < 0.25
C
C2CGP GSEA
Li Estrogene early E2 response pathway
siNT
siFOXA1
siPBX1
siFOXA1 + siPBX1
C1
C2
C3
z-score
Replicate
Rep1
Rep2
Rep3
D
siFOXA1 vs siNT
−log10(padj)
ATAC log2FC
−log10(padj)
log2 Fold Change
E
siPBX1 vs siNT
−log10(padj)
ATAC log2FC
−log10(padj)
log2 Fold Change
F
siFOXA + siPBX1 vs siNT
−log10(padj)
−log10(padj)
ATAC log2FC
log2 Fold Change
G
siFOXA1 vs siNT
XBP1
ESR1
FOXP1
IGFBP4
CCND1
−log10(padj)
−log10(padj)
ATAC log2FC
log2 Fold Change
H
siPBX1 vs siNT
POLR2A
SMARCC2
NFYC
KRT8
CCND1
ATAC log2FC
−log10(padj)
−log10(padj)
log2 Fold Change
I
siFOXA1 + siPBX1 vs siNT
FOXP1
XBP1
IGFBP4
ESR1
CCND1
−log10(padj)
−log10(padj)
ATAC log2FC
log2 Fold Change

Figure S8

Figure S8. Integrated RNA-seq and ATAC-seq Analyses Reveal Concordant Transcriptional and Chromatin Accessibility Changes upon *FOXA1* and *PBX1* Depletion.
(A) MCF7 cells were transfected with *FOXA1* siRNAs and *PBX1* siRNAs either individually or in combination. Forty-eight hours post-transfection, cells were harvested for immunoblotting. s.e., short exposure; l.e., long exposure.
(B) Gene set enrichment analysis (GSEA) across comparisons (si*FOXA1* vs siNT, si*PBX1* vs siNT). FDR < 0.25; NES, normalized enrichment score.
(C) Heatmap of genes from the C2CGP Li Estrogene Early E2 Response gene set (MSigDB) across conditions (siNT, si*FOXA1*, si*PBX1*, and combination; n = 3 biological replicates).
(D-F) Waterfall plots showing the top 50 genes with increased expression associated with gains in chromatin accessibility across si*FOXA1* vs siNT (D), si*PBX1* vs siNT (E), and si*FOXA1* + si*PBX1* vs siNT (F) in full media conditions.
(G-I) Waterfall plots showing the top 50 genes with decreased expression associated with loss of chromatin accessibility across si*FOXA1* vs siNT (G), si*PBX1* vs siNT (H), and si*FOXA1* + si*PBX1* vs siNT (I) in full media conditions.
In the waterfall plots, the bars represent gene expression from RNA-seq datasets correlating with -log10(padj) and each bubble represents a peak whose size correlates with -log10 (padj) and color denotes $\log_2$ fold change.

SUPPLEMENTARY METHODS

Sample Preparation for Mass Spectrometry

Regular IP

The eluted protein samples were reduced using 2.5 µL of 0.2 M DTT for 1 h at 57 °C. The proteins were then alkylated with 2.5 µL of 0.5 M iodoacetamide (Sigma) for 45 min at RT in the dark. The proteins were then digested using 500ng of sequencing grade modified trypsin (Promega) with shaking at room temperature. Peptides were then loaded onto an equilibrated C18 Spin Column (Thermo Fisher Scientific). Peptides were washed three times with 0.1% TFA and spun on the centrifuge. Subsequent washes were done using 0.5% acetic acid and the peptides were then eluted three times using 80% acetonitrile in 0.5% acetic acid. The organic solvent was removed using a SpeedVac concentrator and the sample reconstituted in 0.5% acetic acid.

TurboID

The eluted protein samples were reduced using 2 µL of 0.2 M DTT for 1 h at 57 °C. The proteins were then alkylated with 2 µL of 0.5 M iodoacetamide (Sigma) for 45 min at RT in the dark. Samples were acidified with 12% phosphoric acid to 1.2%. Samples were then diluted with the S-Trap binding buffer containing (90% aqueous methanol containing a final concentration of 100 mM TEAB) and loaded onto the S-Trap (Protifi) which is placed in a 2 mL Eppendorf tube. The samples were then spun at 4,000 g for 1 min. Next, samples were then subsequently washed 3X using the S-Trap binding buffer with the spin step repeated after each wash addition. The S-trap column was then transferred to a new 1.5 mL Eppendorf tube and the proteins were then digested with 1 µg Trypsin (Promega) at 47 °C for 1 h. Peptides from the project containing the empty vector, FOXA1 and RAF1 samples were eluted by the addition of 40% acetonitrile in 0.5% acetic acid, followed by 80% acetonitrile in 0.5% acetic acid. The Other two projects containing CDK4, CDK6, IRF5, and STAT6 samples used an updated S-Trap elution protocol where the peptides were eluted once using 50mM TEAB in water pH 8.5, once using 0.2% formic acid in water, and once using 50% acetonitrile in water. Once all elution steps were complete, the organic solvent was removed using a SpeedVac concentrator and the sample reconstituted in 0.5% acetic acid.

DSS & IP

The eluted protein samples were reduced using 2.5 µL of 0.2 M DTT for 1 h at 57 °C. The proteins were then alkylated with 2.5 µL of 0.5 M iodoacetamide (Sigma) for 45 min at RT in the dark. The proteins were then digested using 500 ng of sequencing grade modified trypsin (Promega) with shaking at RT. Peptides were then acidified to 0.5% TFA using 10% TFA and loaded onto an equilibrated C18 Spin Column (Thermo Fisher Scientific). Peptides were washed three times with 0.1% TFA and spun on the centrifuge. Subsequent washes were done using 0.5% acetic acid and the peptides were then eluted three times using 80% acetonitrile in 0.5% acetic acid. The organic solvent was removed using a SpeedVac concentrator and the sample reconstituted in 0.5% acetic acid.

FA & IP

The samples were incubated at 90 °C for 10 min to remove the formaldehyde. The eluted protein samples were reduced with 2.5 µL of 0.2 M DTT for 1 h at 57 °C. The proteins were then alkylated with 2.5 µL of 0.5 M iodoacetamide (Sigma) for 45 min at RT in the dark. The proteins were then digested using 500 ng of sequencing grade modified trypsin (Promega) with shaking at RT. Peptides were then loaded onto an equilibrated C18 Spin Column (Thermo Fisher Scientific). Peptides were washed three times with 0.1% TFA and spun on the centrifuge. Subsequent washes were done using 0.5% acetic acid and the peptides were then eluted three times using

80% acetonitrile in 0.5% acetic acid. The organic solvent was removed using a SpeedVac concentrator and the sample reconstituted in 0.5% acetic acid.

LC-MS^2 Analysis

For every purification method (MethodGroup1: Regular IP, TurboID; MethodGroup2: DSS, 0,05% FA and 1% FA), an aliquot of each sample was loaded onto a trap column (Acclaim PepMap 100 pre-column, 75 µm × 2 cm, C18, 3 µm, 100 Å, Thermo Fisher Scientific) connected to an analytical column (EASY-Spray column, 50 m × 75 µm internal diameter, PepMap RSLC C18, 2 µm, 100 Å, Thermo Fisher Scientific) using the autosampler an Easy nLC 1000 for MethodGroup1 and an Easy nLC 1200 for MethodGroup2 (Thermo Fisher Scientific). Solvent A consisting of 2% acetonitrile in 0.5% acetic acid and solvent B consisting of 80% acetonitrile in 0.5% acetic acid. The peptide mixture was gradient eluted using the following gradient: 5% solvent B for 5 min, 5-35% solvent B in 60 min, 35-45% solvent B in 10 min, followed by 45-100% solvent B in 10 min. The samples were acquired for MethodGroup1 on the Q-Exactive and for MethodGroup2 on the Orbitrap Eclipse mass spectrometers (Thermo Fisher Scientific) with the following parameters: full MS spectra resolution of 70,000 (MethodGroup1) and 120,000 (MethodGroup2), an AGC target of 1e6 and 4e5 and maximum ion time of 120 and 50 ms, respectively. Scan range from 400 to 1,500 m/z for both groups. The MS/MS spectra were collected using a top 20 data dependent high resolution HCD method for MethodGroup1 and HCD activation for MethodGroup2. Corresponding parameters were: resolution of 17,500 and 30,000, an AGC target of 5e4 and 2e5, maximum ion time of 120 and 200 ms, respectively. Both groups used one microscan, 2 m/z isolation window, normalized collision energy (NCE) of 27 and a dynamic exclusion of 30 s. A first fixed mass of 150 m/z was applied only for MethodGroup1. To identify binding partners, all acquired MS2 spectra were searched against a UniProt human database using Sequest HT within Proteome Discoverer 1.4 (Thermo Fisher Scientific). Fixed modifications were set on cysteine (carbamidomethyl), variable modifications on methionine, and deamidation on glutamine and asparagine. The resulting peptide spectra matches and proteins are filtered to better than 1% false discovery rate (FDR) and only proteins with at least two different peptides are reported. AP-MS data were analyzed using SAINTExpress analysis via the REPRINT web platform (CRAPome 2.0, https://reprint-apms.org/), comparing between the baits and the empty vector to determine interacting proteins. Common contaminants were marked with a “Cont_” tag and subsequently excluded from all downstream analyses.
